# P-284. Effect of Different Disinfection Methods on Hospital Sink Drain Disinfection

**DOI:** 10.1093/ofid/ofae631.487

**Published:** 2025-01-29

**Authors:** Ibrahim Ahmed El-Imam, Lyndsay M O’Hara, Christopher McClintock, Indira French, Gwen Paszkiewicz, Gwen Robinson, Amber R Thomas, J Kristie Johnson, Surbhi Leekha

**Affiliations:** University of Maryland Baltimore, Baltimore, Maryland; University of Maryland School of Medicine, Baltimore, Maryland; University of Maryland Medical Center, Baltimore, Maryland; University of Maryland School of Medicine, Baltimore, Maryland; University of Maryland School of Medicine, Baltimore, Maryland; University of Maryland, Baltimore, Baltimore, Maryland; University of Maryland Medical Center, Baltimore, Maryland; University of Maryland School of Medicine, Baltimore, Maryland; University of Maryland School of Medicine, Baltimore, Maryland

## Abstract

**Background:**

A commercially available foaming disinfectant containing hydrogen peroxide, peracetic acid, and octanoic acid is EPA-registered for biofilm disinfection in sink drains, but recolonization occurs quickly. We evaluated the impact of adding physical removal of bioburden to foaming disinfectant use on bacterial colonization in sink drains.

**Figure:**
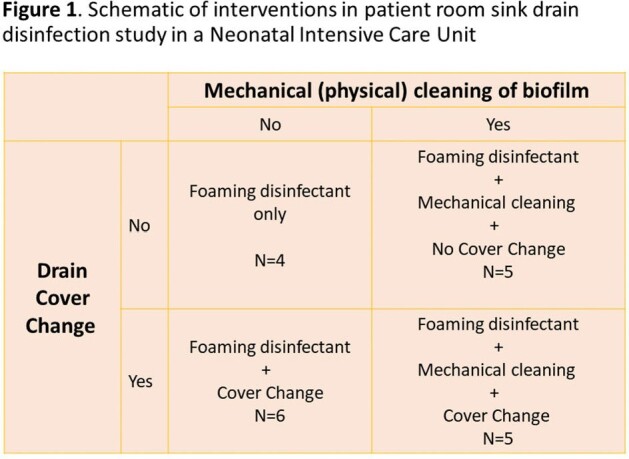

**Methods:**

We conducted a longitudinal study in a level-IV neonatal intensive care unit (NICU) at an academic medical center in Baltimore, MD. The NICU has 52 single-patient rooms with sinks with removable drain plug covers. We included 20 unique rooms to investigate the effect of foaming disinfectant alone and coupled with physical removal of biofilm through (1) mechanical cleaning with a brush, and (2) replacement of drain cover (Figure 1). In addition, we sampled 10 room sinks as control (no intervention). Samples were collected from 2 inches below the sink drain and underside of the drain cover using a polyester tripped swab (Puritan ESK environmental sampling kit with neutralizing buffer) at baseline (day 0) and days 1, 3, 5, and 7 post-intervention. Samples were serially diluted, plated on MacConkey II agar (Becton Dickinson), and incubated for 24 hours at 35+2°C. Colony-forming units (CFU) of total gram-negative bacilli (GNB) were calculated. The primary outcome was the difference in GNB burden (mean log CFUs) from baseline at each time point, for each intervention strategy, compared using paired t-test.

**Figure:**
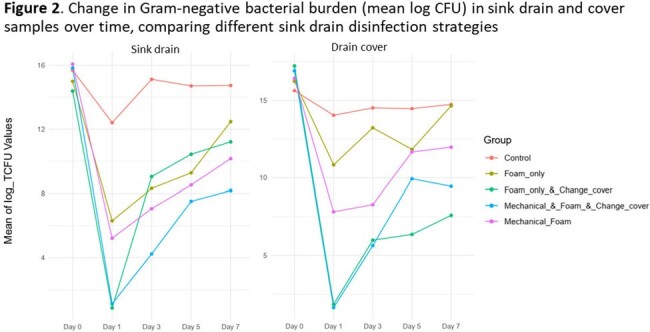

**Results:**

Baseline counts for all groups including control were similar. On day 1, we found significant reductions from baseline for each intervention strategy, with differences from baseline becoming smaller over time. There was no significant change in GNB counts for the control group at any time point (Figure 2, Table 1). Strategies with mechanical cleaning (vs without) performed better for sinks, whereas sink cover change (vs without) resulted in greater log reductions in the cover bioburden through day 7 (Figure 2, Table 1).

**Figure:**
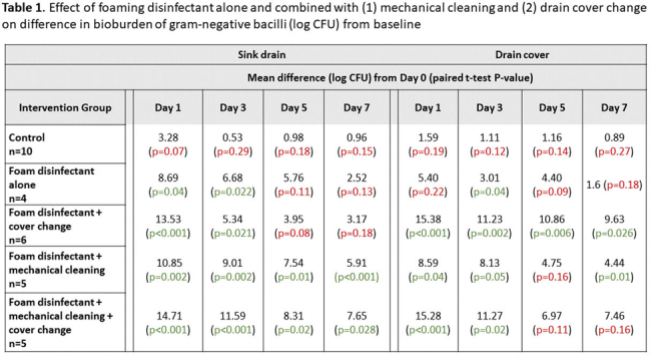

**Conclusion:**

Physical bioburden removal improved the effect of a foaming disinfectant on GNB counts in hospital sink drains. The impact of this strategy on hospital-acquired infections needs to be studied.

**Disclosures:**

**J Kristie Johnson, PhD, D (ABMM)**, biomerieux: has given presentations/speeches for the company listed above

